# Multiple Category-Lot Quality Assurance Sampling: A New Classification System with Application to Schistosomiasis Control

**DOI:** 10.1371/journal.pntd.0001806

**Published:** 2012-09-06

**Authors:** Casey Olives, Joseph J. Valadez, Simon J. Brooker, Marcello Pagano

**Affiliations:** 1 Department of Biostatistics, University of Washington, Seattle, Washington, United States of America; 2 Department of Biostatistics, Harvard University, Boston, Massachusetts, United States of America; 3 Department of International Health, Liverpool School of Tropical Medicine, Liverpool, United Kingdom; 4 Faculty of Infectious and Tropical Diseases, London School of Hygiene and Tropical Medicine, London, United Kingdom; 5 Kenya Medical Research Institute-Wellcome Trust Research Programme, Nairobi, Kenya; Liverpool School of Tropical Medicine, United Kingdom

## Abstract

**Background:**

Originally a binary classifier, Lot Quality Assurance Sampling (LQAS) has proven to be a useful tool for classification of the prevalence of *Schistosoma mansoni* into multiple categories (≤10%, >10 and <50%, ≥50%), and semi-curtailed sampling has been shown to effectively reduce the number of observations needed to reach a decision. To date the statistical underpinnings for Multiple Category-LQAS (MC-LQAS) have not received full treatment. We explore the analytical properties of MC-LQAS, and validate its use for the classification of *S. mansoni* prevalence in multiple settings in East Africa.

**Methodology:**

We outline MC-LQAS design principles and formulae for operating characteristic curves. In addition, we derive the average sample number for MC-LQAS when utilizing semi-curtailed sampling and introduce curtailed sampling in this setting. We also assess the performance of MC-LQAS designs with maximum sample sizes of *n* = 15 and *n* = 25 via a weighted kappa-statistic using *S. mansoni* data collected in 388 schools from four studies in East Africa.

**Principle Findings:**

Overall performance of MC-LQAS classification was high (kappa-statistic of 0.87). In three of the studies, the kappa-statistic for a design with *n* = 15 was greater than 0.75. In the fourth study, where these designs performed poorly (kappa-statistic less than 0.50), the majority of observations fell in regions where potential error is known to be high. Employment of semi-curtailed and curtailed sampling further reduced the sample size by as many as 0.5 and 3.5 observations per school, respectively, without increasing classification error.

**Conclusion/Significance:**

This work provides the needed analytics to understand the properties of MC-LQAS for assessing the prevalance of *S. mansoni* and shows that in most settings a sample size of 15 children provides a reliable classification of schools.

## Author Summary

The control of schistosomiasis calls for rapid and reliable classification tools. This study evaluates the performance of one such tool, Lot Quality Assurance Sampling (LQAS) for assessing the prevalence of *S. mansoni* in African schoolchildren. We outline the design considerations and introduce novel sequential sampling plans for Multiple Category-LQAS. We use data from 388 schools in Uganda, Kenya, and Tanzania to assess the performance of LQAS as a tool for classification of S. mansoni infection into one of three classes: ≤10% >10 and <50%, ≥50%. Our findings suggest that an LQAS-based multiple classification system performs as well as the World Health Organization recommended methods at a fraction of the sampling effort. Our work validates LQAS as a rapid assessment tool and extends it to allow investigators to apply the method to control other infectious diseases.

## Introduction

Schistosomiasis is a tropical disease caused by infection with *Schistosoma* parasitic worms. The disease burden of schistosomiasis is greatest in sub-Saharan Africa (SSA) which shoulders 85% of the global burden [1], [2], with school-age children as well as adolescent girls and women of childbearing age suffering the greatest consequences of infection [3], [4]. The two main species responsible for schistosomiasis in SSA are *Schistosoma haematobium*, which causes urinary schistosomiasis, and *S. mansoni*, responsible for intestinal schistosomiasis.

The World Health Organization (WHO) recommends a three-way classification (≤10%, >10 and <50%, ≥50%) of the prevalence of schistosome infection to determine appropriate interventions for school-age children [4], [5]. These classifications are generally made using classical statistical approaches with data collected in parasitological surveys of between 250 and 500 children in five to ten schools per ecological zone (about 50 children per school) [6], [7]. However, this recommendation is based on logistical concerns more so than statistical ones. Sampling 50 children in multiple schools may be financially prohibitive and there is a need for rapid assesment methods for defining the distribution of infection in order to target control [8]. For identifying communities/schools with high prevalences of *S. haematobium* the WHO recommends the use of questionnaires of self-reported blood in urine or parasitological tests [9]. Concerns about the lack of a reliable questionnaire approach for *S. mansoni* has prompted researchers to explore alternative ways, including the use of lot quality assurance sampling (LQAS), to reduce the sampling effort required to assess the prevalence and distribution of *S. mansoni* based on parasitological surveys [10], [11].

A classification tool, LQAS has been used in a variety of settings to identify program areas as either “acceptable” or “unacceptable” with respect to a preestablished target [12], [13]. As early as 2001, Rabarijaona *et al* utilized LQAS to classify the prevalence of *S. mansoni* [14], [15]. More recently, a number of studies have been published which utilize LQAS to provide a three-way classification of disease. In 2003, Myatt *et al* used LQAS to provide a ternary classification of the prevalence of active trachoma in Malawi [16]. In order to provide a finer classification, Myatt specified two classic LQAS sampling plans with the goal of classifying areas as low/not low and high/not high. Areas classified as both “not low” and “not high” were classified as moderate. In addition, Myatt *et al* allowed for early stopping in the sampling procedure contigent upon reaching a maximum allowable number of failures in the sample. Brooker *et al* went on to apply this three-way LQAS to identify schools with a high prevalence of *S. mansoni* in Uganda, finding that an LQA sample size fo 15 (*n = 15*) provided reliable classification of infection prevalence [8]. This study showed that the use of an LQAS-based classification system in high transmission settings could drastically reduce the cost of treatment when compared both to the conventional survey method and to blanket treatment without prior screening. These studies demonstrate that LQAS can be used to provide a more precise classification than the traditional method.

The Brooker *et al* multiple classification scheme was evaluated by simulation from a large database of 202 Ugandan schools. While this represents an important step toward validating 3-way LQAS, the results are entirely contingent upon the data in the database and therefore provide little insight into how the same method will perform when applied to different regions with even minor deviations in the underlying distribution of disease. Moreover, the simulation approach to validation gives little guidance for designing a survey where prior information is either sparse or unavailable. If a three-way classification LQAS system is to be used in other settings, it is important to understand the statistical underpinnings of the methodology and provide guidelines for designing such surveys in other settings or for other diseases.

The primary aim of the current work is the development of a unified Multiple Category-LQAS tool (MC-LQAS) and the validatation of its use in multiple setting in East Africa. Specifically, we outline the theoretical underpinnings of MC-LQAS system, focusing on classification into one of three categories, and provide guidelines for choosing design parameters. Next, we present the theoretical aspects of sequential sampling as employed by researchers in the field [8], [16], [17], [18]. Sequential LQAS, known in the statistical literature as semi-curtailed sampling, allows for potential reduction in the sample size required to make a decision without impacting classification error. A worked example of the tool to assess the prevalence of *S. mansoni* in 388 schools in Kenya, Uganda, and Tanzania is given [19], [20], [21]. The primary design examined is that used by Brooker *et al* [8] and we discuss the analytical properties of this approach. Finally, we validate the multiple classification system against the standard approach to classification and show that the use of an LQAS based system can substantially reduce the necessary sample size, while providing valid information for selecting the appropriate intervention strategy.

## Methods

### LQAS

**Figure 1:**
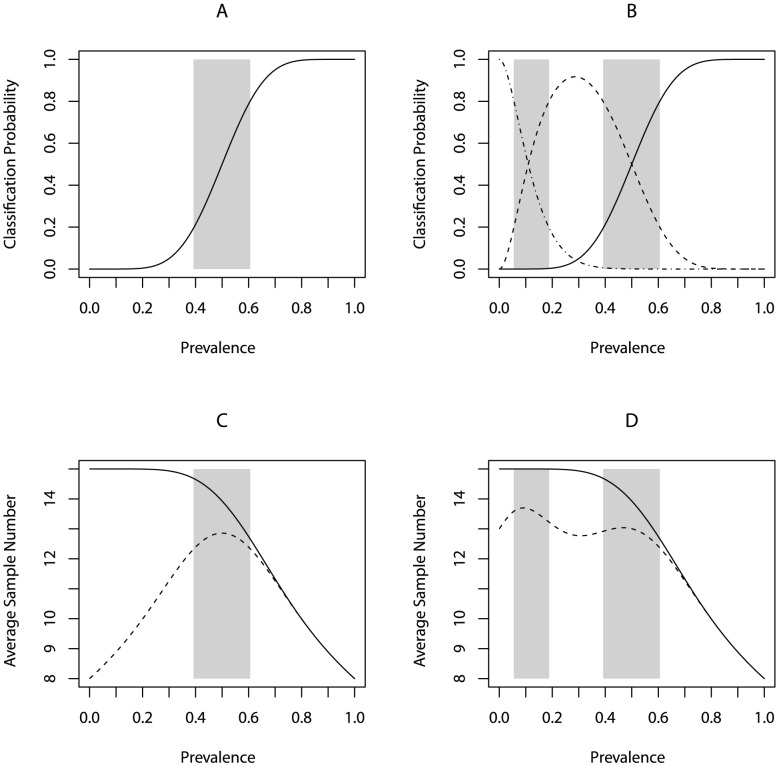
Operating Characteristic curves and Average Sample Number for LQAS and MC-LQAS. A) Operating Characteristic curve for LQAS design with n = 15,d = 7, and α = β = 0.20; B) Operating Characteristic curves for low (dashed-dotted), medium (dashed), and high (solid) categories for MC-LQAS design with n = 15, d_1_ = 1, d_2_ = 7, and δ_1_ = δ_1_ = δ_1_ = δ_1_ = 0.20; C) Average Sample Number for semi-curtailed (solid) and curtailed (dashed) LQAS with n = 15 and d = 7; D) Average Sample Number for semi-curtailed (solid) and curtailed (dashed) MC-LQAS with n = 15, d_1_ = 1, and d_2_ = 7.

Traditional LQAS calls for a random sample of *n* binary observations from a “lot”. If the number of successes in the sample, *X*, is less than or equal to a predefined decision rule, *d*, the locale is classified as unacceptable. Otherwise, the locale is classified as acceptable. The word “success” is a statistical convention but typically denotes a failure to meet an established criterion or receive an intervention. In the case of sampling for *S. mansoni*, the number of successes are cases of *S. mansoni* infection. If the number of infected cases exceeded a pre-determined level, then the lot is rejected and the school/community is identified as in need of mass treatment. A succinct summary of any LQAS design is the Operating Characteristic (OC) curve [22]. The OC curve depicts the probability of an acceptable classification against the true underlying prevalence, *p*. We assume that *p* represents the proportion in a given population with infection, such as *S. mansoni* infection. An example of an OC curve is given in Figure 1A with *n = 15* and *d = 7*.

The choice of the sample size and decision rule are critical, as they determine the expected classification error in the procedure. Generally, *n* and *d* are chosen so that the probability of incorrectly classifying a locale as having low prevalence is less than or equal to *α* and the probability of incorrectly classifying a locale as having high prevalence is less than or equal to *β*. In many cases, practitioners associate the labels “low” and “high” with values of the prevalence below and above some value, p*, respectively. In practice, classification probabilitites are evaluated at upper and lower thresholds *p_U_* and *p_L_*, and the value of p* serves little purpose aside from informing the choice of these two parameters. For an appropiately chosen design, the values of the OC curve at *p = p_U_* and *p = p_L_* will be approximately equal to some predefined values 1−*α* and *β*, respectively. For example, the design depicted in Figure 1A is chosen so that at *p_L_ = 0.40* and *p_U_ = 0.60*, the probability of an acceptable classification is less than or equal to *β = 0.20* to the left of *p_L_* and greater than or equal to *1−α = 0.80*, to the right of *p_U_*.

Due to the monotonicity of the OC curve, it follows that for any *p* beyond the upper or lower thresholds, the probability of committing an error is no greater than *α* or β.
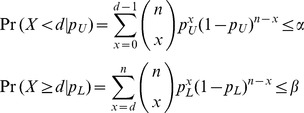
An additional property of the OC curve is that it makes explicit the values of *p* for which LQAS runs a risk that is higher than the maximum of α and β; the value of the OC curve increases from *β* to *1−α* as *p* increases from *p_L_* to *p_U_*. The area between *p_L_* and *p_U_* is commonly referred to as the “grey region”. Thus a locale which truly has prevalence *p* such that *p_L_<p<p_U_* will be classified as acceptable with probability somewhere in the range (*β, 1−α*) (assuming *β<1−α*).

### MC-LQAS

The MC-LQAS procedure extends basic LQAS by classifying a sample against multiple decision rules. In the following we develop MC-LQAS for three-way classification although the method is generalizable to more than three categories. For three-way classification, we must choose a total of two decision rules, *d_1_* and *d_2_*. If the number of successes, *X*, out of a total of *n* observations is less than or equal to *d_1_*, classify the prevalence as low. If *X* is greater than *d_2_*, classify the prevalence as high. Otherwise, classify the prevalence as medium, the middle category.

Analogous to the OC curve, for a specific design we can plot the probability of classification into each of the three categories against *p* to succinctly summarize the MC-LQAS design. Figure 1B shows the OC curve for a three-way classification procedure with *n = 15, d_1_ = 1, d_2_ = 7*. Note that this is a simple extension of the two-way design discussed previously where we now allow for the “unacceptable” category to be parsed into “moderate” and “low”, thus making explicit the connection between this development and that of Myatt *et al* and Brooker *et al* [8], [16]. Of note is the bell-shape of the curve for classification into the moderate category. The lack of monotonicity for this curve is one characteristic of MC-LQAS which sets it apart from LQAS and plays an important role with respect to choosing a design.

As with LQAS, in practice we choose to control for potential misclassification at predetermined thresholds, which we call *p_L1_*, *p_U1_*, *p_L2_*, and *p_U2_*. These should be chosen so that p_L1_<p_1_*<p_U1_ and p_L2_<p_2_*<p_U2_, and in practice it oftentimes makes sense to set p_1_* = p_L1_ and p_2_* = p_U2_. To control for the amount of misclassification, we choose *d_1_* and *d_2_* so that the probability of *correct* classification remains high at these thresholds. That is, choose the decision rules so that
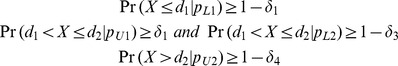
where *δ_1_*, *δ_2_*, *δ_3_* and *δ_4_* reflect the acceptable levels of potential error determined by the investigator. This is directly analogous to choosing upper and lower thresholds, *p_L_* and *p_U_*, in classical two-way LQAS with the notable exception of the moderate category, where we see that it is important to control for the possible error at two locations. This has to do with the aforementioned bell shape of the moderate OC curve. The lack of monotonicity makes it so that one must control for error at both *p_U1_* and *p_L2_*.

We note that in the above formulation, we have ignored possible misclassification into the extreme categories. Depending on the distance between thresholds, misclassification into a non-contiguous category can be minimal for even small samples. Hence, for moderate sample sizes, we only worry about four possible misclassifications, which are those misclassifications into contiguous classes.

### Curtailed and Semi-Curtailed Designs

In certain situations, it is possible to reduce the sample size needed to reach a decision by “sampling to the decision rule”. For example, suppose we define a traditional LQAS plan with a sample size *n = 15* and *d = 7*. Suppose further that during data collection we find that the first eight observations are successes. At this point, we need not sample further to know the resulting classification will be acceptable. The analytical properties of this type of sampling are neither well-documented nor well-understood in the public health literature. However, this process is referred to as *semi-curtailed* sampling in the statistics literature where it has been in use for the past fifty years [23], [24], [25]. The main benefit of this type of sampling is the potential to reduce the overall number of observations, or the Average Sample Number (ASN), required to reach a decision.The semi-curtailed ASN is plotted as a function of the prevalence with *n = 15* and *d = 7* in Figure 1C and its derivation provided in the Appendix S1. A feature of semi-curtailed sampling is that it preserves the OC curve, which means that the expected error rates are not affected [25]. Thus, there seems to be little drawback to employing semi-curtailed sampling when feasible to reduce the sample size.

Indeed, one can benefit even more by adopting a *curtailed* sampling plan [23]. That is, one can terminate sampling either if the number of successes is too great or too few at a given point. To continue with our example, suppose instead that the first eight observations are failures. In this case, it is not possible to observe more than seven successes in the remaining observations, and sampling can also cease. The curtailed ASN plotted as a function of the prevalence with *n = 15* and *d = 7* is plotted in Figure 1C and its derivation is included in the Appendix S1. Once again, the employment of curtailed sampling does not affect the OC curve.

The notion of curtailed sampling is easily extended to MC-LQAS. For example, MC-LQAS also allows for the potential of early stopping by sampling to the decision rule, or semi-curtailed sampling. For example, when utilizing an MC-LQAS design with *n = 15*, *d_1_ = 1* and *d_2_ = 7*, sampling can terminate with a high classification as soon as the number of successes excedes seven. The ASN for MC-LQAS when employing semi-curtailed sampling is equal to the ASN in traditional LQAS.

The curtailed version of MC-LQAS is slightly different than its traditional counterpart in that it allows for early stopping for low, moderate, or high classifications. Continuing with our example, if the first thirteen observations are failures, then it follows that the lot will be classified as low irrespective of the remaining observations. Likewise, if in the first twelve observations are four successes and eight failures, then sampling can stop with a moderate classification, as neither low nor high classifications are possible at this point. The semi-curtailed and curtailed ASNs for an MC-LQAS design with *n = 15*, *d_1_ = 1*, and *d_2_ = 7* are plotted as a function of the prevalence in Figure 1D. We note that the functional form of the ASN under curtailed sampling will generally be bi-model, which reflects the two areas of uncertainy or grey regions. It follows for the same reasons as in the traditional LQAS setting that the OC curves for MC-LQAS are not affected by sequential sampling of this nature. Proofs of these results are given in the Appendix S1.

### Application to Prevalence of *S. mansoni* in East African Schools

**Figure 2:**
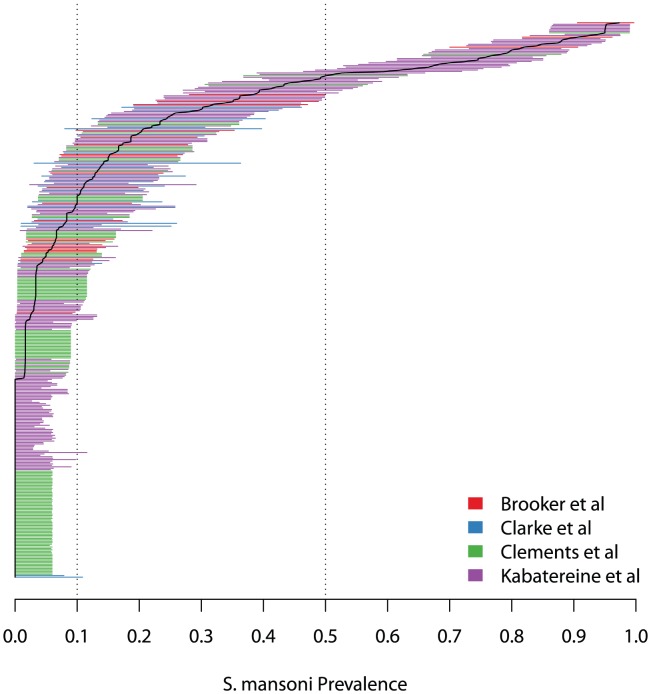
Prevalence of *S. mansoni* in 388 East African schools. Horizontal lines are 95% exact confidence intervals.

In the following, we consider *S. mansoni* data reported in four different studies; two in Kenya [19], [21], one in Uganda [26], and one in Tanzania [20]. In each study, a sample of school children in multiple schools were randomly selected to provide stool samples which were examined microscopically for the ova of *S. mansoni*, hookworm, *Ascaris lumbricoides*, and *Trichuris trichiura*. The number of schools sampled ranges from 21 [19] study to 199 [26] with school sample sizes as low as 21 and as high as 202. In Figure 2, the estimated prevalence of *S. mansoni* in each of the 388 schools, along with 95% exact binomial confidence intervals, are plotted for each of the four studies.

We use these data to assess the performance of the MC-LQAS design with *n = 15*, *d_1_ = 1*, and *d_2_ = 7* and compare with expected performance. This design differs slightly from that which was utilized in the 2005 Brooker study, where *d_1_ = 2* [8]. Our current choice reflects a 2006 change in WHO guidelines which shifted the lower programmatic threshold from 20% to 10% [5]. Note that decision rules *d_1_ = 1* and *d_2_ = 7* corresponds to prevalence decision thresholds of 6.7% and 46.7%, respectively. To choose upper and lower thresholds, we assume that the desired probability of correct classification should be greater than or equal to 0.80 uniformly (i.e. *δ_1_ = δ_2_ = δ_3_ = δ_4_ = 0.20*). Under this assumption, we can solve for the upper and lower thresholds, yielding *p_L1_ = 0.055*, *p_U1_ = 0.188*, *p_L2_ = 0.392*, and *p_U2_ = 0.606*. Additionally, to assess the impact of increasing the sample size on classification agreement, we consider an MC-LQAS design with *n = 25*, *d_1_ = 2* and *d_2_ = 12*. Using the same approach, we identified upper and lower thresholds of *p_L1_ = 0.062*, *p_U1_ = 0.164*, *p_L2_ = 0.417*, and *p_U2_ = 0.583* for this design.

We generate 1000 MC-LQAS classifications of each school in the sample by repeatedly “sampling down” the individual data to 15 or 25 students and classifying each school based on these observations. To compare the classifications resulting from MC-LQAS with those that result from binning the full sample prevalence, we calculate for each simulation the weighted kappa statistic, which measures agreement between classification methods across locations [27]. We report the mean kappa statistic and interquartile range (IQR) across the 1000 simulations. In addition, we calculate the ASN in each simulation when employing both semi-curtailed and curtailed sampling plans and report the mean ASN and IQR across the 1000 simulations. Lastly, we calculate the proportion correctly classified as a function of the full sample prevalence. All simulations were conducted using R statistical software, version 2.11.1 [28].

## Results

**Figure 3:**
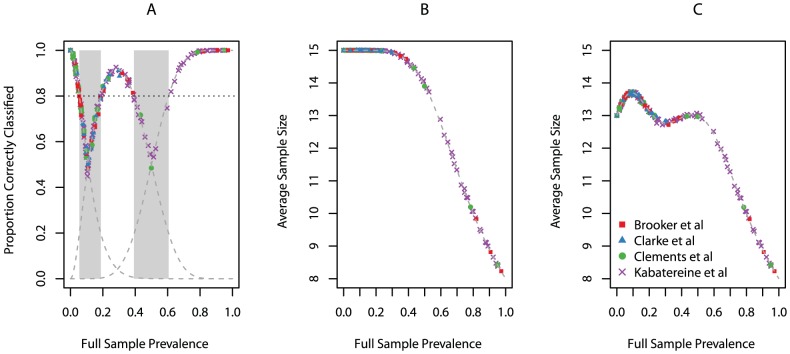
School-based simulation results. For simulation with n = 15, d_1_ = 1, and d_2_ = 7, (A) average proportion of correctly classified schools with expected Operating Characteristic curves overlaid (dashed grey), (B) Average Sample Number when utilizing semi-curtailed sampling with expected Average Sample Number overlaid (dashed grey), and (C) Average Sample Number when utilizing curtailed sampling with expected Average Sample Number overlaid (dashed grey).

Figure 3A displays the average proportion of schools correctly classified as a function of the full sample prevalence. For expository purposes, we overlay the OC curve for this design, noting that the simulation results and expected curves coincide. Likewise, we display the average ASN under semi-curtailed (Figure 3B) and curtailed (Figure 3C) sampling as a function of the full sample prevalence and overlay the expected ASN curves. Once again, these quantities coincide, as expected.

The results of our simulation study are presented in Table 1. The overall agreement between the MC-LQAS with a sample size of 15 and full sample classifications was 0.87 (IQR: 0.86–0.89). Although not everyone agrees on the interpretation of the kappa statistic, values greater than 0.60 are commonly interpretted as implying “substantial” agreement, whereas values greater than 0.80 are thought to imply “almost perfect” agreement. For three of the four studies, the agreement between the MC-LQAS and the full sample classifications was high (*κ>0.75*) [27]. On average the use of the MC-LQAS procedure resulted in either substantial or almost perfect agreement with these data.

**Table 1:** Kappa-statistic, Curtailed Average Sample Number, and Semi-curtailed Average Sample Number in four studies for two different MC-LQAS designs.

		n = 15, d_1_ = 1, d_2_ = 7			n = 25, d_1_ = 2, d_2_ = 12		
	SS (# Schools)	κ-statistic	Curtailed ASN	Semi-Curtailed ASN	κ-statistic	Curtailed ASN	Semi-Curtailed ASN
All Studies	25246 (388)	0.875 (0.864–0.885)	12.902 (12.871–12.936)	14.504 (14.487–14.523)	0.897 (0.888–0.907)	21.538 (21.486–21.593)	24.158 (24.137–24.180)
Brooker et al	1739 (25)	0.774 (0.723–0.830)	12.892 (12.760–13.040)	14.255 (14.200–14.320)	0.822 (0.780–0.874)	21.263 (21.000–21.520)	23.730 (23.640–23.800)
Clarke et al	1093 (21)	0.459 (0.341–0.576)	13.371 (13.190–13.571)	14.991 (15.000–15.000)	0.518 (0.394–0.615)	21.859 (21.524–22.202)	24.997 (25.000–25.000)
Clements et al	8617 (143)	0.763 (0.722–0.808)	13.119 (13.091–13.147)	14.909 (14.895–14.923)	0.808 (0.771–0.844)	22.100 (22.042–22.161)	24.847 (24.832–24.860)
Kabatereine et al	13797 (199)	0.901 (0.889–0.912)	12.698 (12.648–12.749)	14.196 (14.166–14.231)	0.920 (0.910–0.931)	21.139 (21.055–21.216)	23.631 (23.588–23.678)

Point estimates are mean and quantities in parentheses represent the inerquartile range over 1000 simulated datasets.

The notable exception was the Clarke *et al* study from Kenya, where the kappa statistic was 0.46 (IQR: 0.34–0.58) when *n = 15*. Of the four studies, the Clarke *et al* study had the fewest observations and fewest schools. Furthermore, of the 21 schools sampled in this study, 13 schools had full sample prevalence lying within one of the two grey regions where potential error is known to be high (in comparison with 13 of 25, 20 of 143, and 49 of 199 in the Brooker, Clements, and Kabatereine studies, respectively). Thus, this MC-LQAS design is expected to be sub-optimal for this type of underlying distribution of prevalences. One might improve performance by increasing the sample size. The kappa statistics for all studies slightly increased when using a sample of size 25 (Table 1), although agreement in the Clarke study remained low with a kappa statistic of 0.52 (IQR: 0.39–0.62).

The ASN for a maximum sample size of n = 15 when utilizing curtailed and semi-curtailed sampling was 12.90 (IQR: 12.87–12.94) and 14.50 (IQR: 14.49–14.52), respectively. When the maximum sample size was increased to n = 25, the ASN for curtailed and semi-curtailed sampling increased to 21.54 (IQR: 21.50–21.59) and 24.2 (24.14–24.18), respectively.

## Discussion

This work outlines a unified and systematic approach to designing Multiple Category-LQAS classification systems with application to the prevalence of *S. mansoni* in schoolchildren. Through simulation and using real data, we show it performs as well as existing methods in practice for classification of the prevalence of infection at a fraction of the sampling effort. Furthermore, for the first time in the public health literature, we have elucidated the theoretical properties of “sampling to the decision rule”, or semi-curtailed sampling, in LQAS, and extended these notions to multiple classification. Our validation study shows that an MC-LQAS design with *n = 15*, *d_1_ = 1*, and *d_2_ = 7* provides classifications in near perfect agreement with the standard “binning” approach, yet using less than half as many observations. As expected, agreement between MC-LQAS and full sample classifications tends to be the worst for prevalences lying within the grey region (as found in the Clarke study), where the risks of classification error are high.

Our findings resonate with empirical results pointing to the reliability and potential cost-reduction associated with using LQAS for rapid assessment of *S. mansoni* [8]. Recent research suggests that an LQAS-based approach may also perform better than sophisiticated geostatistical modeling strategies with respect to correct classification, although at a higher cost per high prevalence school correctly classified [11]. Thus, while we have shown that MC-LQAS is a reliable tool for classification, investigators should continue to take care to choose the evaluative approach which best suits a given situation.

A limitation of this study is the lack of consideration for diagnostic sensitivity and specificity. The standard method for diagnosis of *S. mansoni* is the Kato-Katz method, which has been criticized for having low sensitivity that varies depending on the intensity of infection in an individual [29], [30]. Some studies have found sensitivities as low as 0.60, which is a serious violation of the perfect diagnostic test assumption. Methods for estimating the prevalence of *S. mansoni* in the presense of variable sensitivity and infection intensity is an area of ongoing research [31].

A shortcoming of our study is that we ignore the underlying distribution of prevalence. In the event that prior information on the level or distribution of *p* is available, Olives and Pagano provide Bayesian methods for choosing the sample size and decision rule for traditional LQAS [32]. Olives discusses the same approach in the context of multiple classification in [33], providing the basis for incorporating complex disease dynamics into the model. Although ignoring prior information does not impact the viability of our results, it is expected that incorporating this extra information would improve expected performance.

A strength of our study is the principled treatment of curtailed and semi-curtailed sampling in LQAS. The ASN is a largely ignored piece of information that program managers can utilize to inform their choice of LQAS design. Note that curtailed sampling plans allow for early stopping with a classification of moderate prevalence, in addition to low and high. This is in contrast to other sequential LQAS designs used for multiple category classification in the literature, such as those used to classify transmitted HIV drug resistance [34]. In the context of the classification of *S. mansoni* prevalence, the use of curtailed designs will ultimately require fewer stool samples to be analyzed via microscopy. The reduction in sample size will be most pronounced in high prevalence schools, where as few as eight slides may need to be read before reaching a decision. Unfortunately in many cases, slides will be prepared for all participants and sent to the laboratory for microscopic inspection. Thus these savings are likely to be less pronounced in the field than in the laboratory. For other diseases, such as malaria and urinary schistosomiasis, where rapid diagnositc tests and dipsticks (for haematuria) are the modes of diagnosis, the use of curtailed sampling may be of more importance in the field.

Further work is required to evaluate the use of MC-LQAS for sampling for several infections; for example the collection of stool samples to diagnose *S. mansoni* infection and urine samples to diagnose *S. haematobium*, using either dipsticks for the detection of haematruia or the urine filatration technique. How such an integrated approach compares to the use of questionnaire surveys for *S. haematobium* also needs to be investigated.

LQAS as a tool has come to be associated with simplicity and versatility. MC-LQAS maintains these attributes so as to be useful to a wider audience of practitioners. Here we consider the case of *S. mansoni*, and show that as a tool for classification of the prevalence of infection, MC-LQAS is both reliable and adapatable. However, just as LQAS has had extensive use in multiple areas in health, we anticipate that this work will have implications reaching well beyond schistosomiasis for other infectious diseases, such as malaria. The design we describe allows for easy adaptation to other circumstances.

## Supporting Information

Appendix S1 **Derivations of ASN and preservation of OC curves under curtailed and semi-curtailed sampling for MC-LQAS.**
(DOCX)
