# Supplementary material for: Multiple Category-Lot Quality Assurance Sampling: A New Classification System with Application to Schistosomiasis Control
Source: PLoS Negl Trop Dis. 2012 Sep 6;6(9):e1806. doi: 10.1371/journal.pntd.0001806 (PMC3435238; doi:10.1371/journal.pntd.0001806)
Supplement: Appendix S1 — Derivations of ASN and preservation of OC curves under curtailed and semi-curtailed sampling for MC-LQAS. (DOCX) [file pntd.0001806.s001.docx]

**Appendix S1**

**Derivation of Curtailed and Semi-Curtailed ASN for MC-LQAS**

Let *R* be the number of observations required to classify a sample of maximal size *n* as either low, moderate, or high in a population with prevalence *p*.

*Semi-Curtailed Sampling*

Consider first the case of semi-curtailed sampling. Here, *R* takes on the value *n* if the lot is declared “low” or “moderate'', and the value *r=d_2_+1, …, n* if the lot is declared “high”. Letting $F_{n}(\cdot)$ be the cumulative distribution function of a binomial random variable with sample size *n* and probability *p*, it follows that

$$\begin{matrix} E[R]=nPr(X\leq d_{2})+\sum_{r=d_{2}+1}^{n} rPr(R=r)=nF_{n}(d_{2})+\sum_{r=d_{2}+1}^{n} r\left(\begin{matrix} r-1 \\ d_{2} \end{matrix}\right)p^{d_{2}+1}(1-p)^{r-d_{2}-1} \\ \text{ =}nF_{n}(d_{2})+\frac{d_{2}+1}{p}\sum_{r=d_{2}+1}^{n} \left(\begin{matrix} r \\ d_{2}+1 \end{matrix}\right)p^{d_{2}+2}(1-p)^{r-d_{2}-1} \\ \text{ =}nF_{n}(d_{2})+\frac{d_{2}+1}{p}(1-F_{n+1}(d_{2}+1)), \end{matrix}$$

where the last step follows by the following identity due to Patil [1]

$\sum_{y=k}^{n} \left(\begin{matrix} y-1 \\ k-1 \end{matrix}\right)p^{k}(1-p)^{y-k}=\sum_{x=k}^{n} \left(\begin{matrix} n \\ x \end{matrix}\right)p^{x}(1-p)^{n-x}$.

*Curtailed Sampling*

The proof for fully curtailed sampling relies on similar elements. We have that

$E[R]=\sum_{r=0}^{d_{1}} rPr(R=r)+\sum_{r=d_{1}+1}^{d_{2}} rPr(R=r)+\sum_{r=d_{2}+1}^{n} rPr(R=r)$.

For the same reasons as above, it follows that the third piece of this expression is given by

$\sum_{r=d_{2}+1}^{n} rPr(R=r)=\frac{d_{2}+1}{p}(1-F_{n+1}(d_{2}+1))$.

To solve for the first piece, it is convenient to parameterize in terms of the number of failures. Intuitively, we classify the lot as “low” if there are too many failures early on. More formally, we classify a lot as low if the number of successes is greater than or equal to *n-d_1_*. Focusing on the first component and defining *q=1-p*, we have that

$$\begin{matrix} \sum_{r=0}^{d_{1}} rPr(R=r)=\sum_{s=n-d_{1}}^{n} s\left(\begin{matrix} s-1 \\ n-d_{1}-1 \end{matrix}\right)q^{n-d_{1}}(1-q)^{s-(n-d_{1})} \\ \text{ =}\frac{n-d_{1}}{q}\sum_{s=n-d_{1}}^{n} \left(\begin{matrix} s \\ n-d_{1} \end{matrix}\right)q^{n-d_{1}}(1-q)^{s-(n-d_{1})} \\ \text{ =}\frac{n-d_{1}}{q}(1-G_{n+1}(n-d_{1}))\text{ by (1)} \\ =\frac{n-d_{1}}{q}F_{n+1}(d_{1}) \end{matrix}$$

where , which gives us our result. Lastly, to solve for the second component of *E[R],* we note that

$$\sum_{r=d_{1}+1}^{d_{2}} rPr(R=r)=\sum_{r=A+d_{1}+1}^{A+d_{2}} r\left(\begin{matrix} r-1 \\ A-1 \end{matrix}\right)p^{r-A}(1-p)^{A}+\sum_{r=T+n-d_{2}}^{T+n-d_{1}-1} r\left(\begin{matrix} r-1 \\ T-1 \end{matrix}\right)p^{T}(1-p)^{r-T}$$

where *A=n-d_2_* and *T=d_1_+1*. This can be rewritten as

$$\begin{matrix} \left(\sum_{r=0}^{A+d_{2}} r\left(\begin{matrix} r-1 \\ A-1 \end{matrix}\right)p^{r-A}(1-p)^{A}-\sum_{r=0}^{A+d_{1}} r\left(\begin{matrix} r-1 \\ A-1 \end{matrix}\right)p^{r-A}(1-p)^{A}\right) \\ \text{ +}\left(\sum_{r=0}^{T+n-d_{1}-1} r\left(\begin{matrix} r-1 \\ T-1 \end{matrix}\right)p^{T}(1-p)^{r-T}-\sum_{r=0}^{T+n-d_{2}-1} r\left(\begin{matrix} r-1 \\ T-1 \end{matrix}\right)p^{T}(1-p)^{r-T}\right) \end{matrix}$$

We recognize now that each of these quantities can be treated as we did for the first and last components as before, which results in the following derivation

$$\frac{n-d_{2}}{1-p}(F_{n+1}(d_{2}+1)-F_{n-d_{2}+d_{1}+1}(d_{1}+1))+\frac{d_{1}+1}{p}(F_{n-d_{2}+d_{1}+1}(d_{1}+1)-F_{n+1}(d_{1}+1))$$

Combining these results, we have that the average sample number under curtailed sampling for MC-LQAS is given by

$$\begin{matrix} E[R]=\frac{n-d_{1}}{1-p}F_{n+1}(d_{1})+\frac{n-d_{2}}{1-p}(F_{n+1}(d_{2}+1)-F_{n-d_{2}+d_{1}+1}(d_{1}+1)) \\ \text{ +}\frac{d_{1}+1}{p}(F_{n-d_{2}+d_{1}+1}(d_{1}+1)-F_{n+1}(d_{1}+1))+\frac{d_{2}+1}{p}(1-F_{n+1}(d_{2}+1)). \end{matrix}$$

**Preservation of MC-LQAS Operating Characteristic Curves under Curtailed and Semi-Curtailed Sampling**

*Semi-Curtailed Sampling*

Under semi-curtailed sampling, probability of classification into either the “low” or “moderate” classes is not impacted. However, we allow for early stopping due to a “high” classification. In this case, the probability of classification as “high” is given by

$$\sum_{r=d_{2}+1}^{n} \left(\begin{matrix} r-1 \\ d_{2} \end{matrix}\right)p^{d_{2}+1}(1-p)^{r-d_{2}-1}=\sum_{r=d_{2}+1}^{n} \left(\begin{matrix} n \\ r \end{matrix}\right)p^{r}(1-p)^{n-r}$$

which follows by identity (1) above. The right-hand side of this equivalence is recognized as the probability of a high classification under a full sampling plan.

*Curtailed Sampling*

It follows for the same reasons as above that the probability of “high” classification is preserved under curtailed sampling. Thus, it remains to be shown that the probability of a “low” and “moderate” classification is the same under curtailed sampling as it is under the full sampling plan.

We begin by considering the probability of classification in the “low” category. Writing the probability of a low classification in terms of the number of failures, rather than succusses, results in the following,

$$\begin{matrix} \sum_{s=n-d_{1}}^{n} \left(\begin{matrix} s-1 \\ n-d_{1}-1 \end{matrix}\right)q^{n-d_{1}}(1-q)^{s-(n-d_{1})}=\sum_{s=n-d_{1}}^{n} \left(\begin{matrix} n \\ s \end{matrix}\right)q^{s}(1-q)^{n-s}\text{ by (1)} \\ \text{ }=\sum_{s=n-d_{1}}^{n} \left(\begin{matrix} n \\ s \end{matrix}\right)p^{n-s}(1-p)^{s} \\ \text{ =} \sum_{s=0}^{d_{1}} \left(\begin{matrix} n \\ s \end{matrix}\right)(1-p)^{n-s}p^{s}, \end{matrix}$$

the right-hand side of which is recognized as the probability of a “low” classification under full sampling. It follows that classification into the moderate category is preserved by noting that *Pr(Moderate) = 1-Pr(Low) – Pr(High)*.

References

[1] Patil, GP (1960). On the evalutation of the negative binomial distribution with examples. Technometrics 2: 501-505.
